# Utilising sigmoid models to predict the spread of antimicrobial resistance at the country level

**DOI:** 10.2807/1560-7917.ES.2020.25.23.1900387

**Published:** 2020-06-11

**Authors:** Noga Fallach, Yaakov Dickstein, Erez Silberschein, John Turnidge, Elizabeth Temkin, Jonatan Almagor, Yehuda Carmeli, on behalf of the DRIVE-AB Consortium

**Affiliations:** 1 National Institute for Antibiotic Resistance and Infection Control, Tel Aviv Sourasky Medical Center, Tel Aviv, Israel; 2 University of Adelaide, Adelaide, Australia; 3 Sackler School of Medicine, Tel Aviv University, Tel Aviv, Israel; 4 http://drive-ab.eu

**Keywords:** antimicrobial resistance, surveillance, modelling, predictions

## Abstract

**Background:**

The spread of antimicrobial resistance (AMR) is of worldwide concern. Public health policymakers and pharmaceutical companies pursuing antibiotic development require accurate predictions about the future spread of AMR.

**Aim:**

We aimed to identify and model temporal and geographical patterns of AMR spread and to predict future trends based on a slow, intermediate or rapid rise in resistance.

**Methods:**

We obtained data from five antibiotic resistance surveillance projects spanning the years 1997 to 2015. We aggregated the isolate-level or country-level data by country and year to produce country–bacterium–antibiotic class triads. We fitted both linear and sigmoid models to these triads and chose the one with the better fit. For triads that conformed to a sigmoid model, we classified AMR progression into one of three characterising paces: slow, intermediate or fast, based on the sigmoid slope. Within each pace category, average sigmoid models were calculated and validated.

**Results:**

We constructed a database with 51,670 country–year–bacterium–antibiotic observations, grouped into 7,440 country–bacterium–antibiotic triads. A total of 1,037 triads (14%) met the inclusion criteria. Of these, 326 (31.4%) followed a sigmoid (logistic) pattern over time. Among 107 triads for which both sigmoid and linear models could be fit, the sigmoid model was a better fit in 84%. The sigmoid model deviated from observed data by a median of 6.5%; the degree of deviation was related to the pace of spread.

**Conclusion:**

We present a novel method of describing and predicting the spread of antibiotic-resistant organisms.

## Introduction

The increasing prevalence of antimicrobial resistance (AMR) is of worldwide concern [1-3]. Both the World Health Organization (WHO) and the United States (US) Centers for Disease Control and Prevention (CDC) have published lists ranking the threat posed by various resistant pathogens [4,5]. These pathogens have been linked to prolonged hospital stays, greater mortality and higher costs [6-11]. Efforts to control and limit the spread of resistant bacteria and to develop new antimicrobials targeting them are dependent on accurate information about the magnitude of the problem. Policymakers and pharmaceutical corporations looking to invest resources require accurate predictions about the future spread of AMR based on current data [12].

While the magnitude of the problem could be determined using data from surveillance systems (which are currently inadequate) [13], estimating future trends requires modelling. The choice of model type determines in large part the results it will yield, yet most published models of AMR spread provide little justification for model type [14]. Previous models to predict AMR spread assumed a linear increase over time [15] or assumed a 40% increase over current levels [16]. There is evidence that AMR spread tends to follow a sigmoid (logistic) shape [17] i.e. an initial period of low-level resistance followed by a rapid increase in resistance and then a levelling-off at a level less than 100% resistance. Furthermore, many models do not take into account between-country differences in diffusion rates, which may be marked [18]. While many factors affect the pace of resistance spread, any attempt to predict future AMR levels with a ‘one-size-fits-all’ approach risks significant over- or underestimation. In the present article, we aimed to identify and model temporal and geographical patterns of AMR spread and predict future trends based on a slow, intermediate or rapid rise in resistance.

## Methods

### Data sources

We obtained isolate-level data from three antibiotic resistance surveillance projects that were provided to us by their owners: (i) the Australian Enterococcal Sepsis Outcome Programme (AESOP) and Enterobacteriaceae Sepsis Outcome Programme (EnSOP), both from the Australian Group on Antimicrobial Resistance (http://agargroup.org.au), which included blood specimen isolates from the year 2013, (ii) the Tigecycline Evaluation Surveillance Trial (TEST; Pfizer, New York, US), which included blood and respiratory specimens worldwide from 2004 to 2014 and (iii) the Meropenem Yearly Susceptibility Test Information Collection (MYSTIC; AstraZeneca, Cambridge, United Kingdom), which included blood and respiratory specimens worldwide from 1997 to 2007. Each surveillance project included data on the type of bacterium and its susceptibility to at least one antibiotic. Resistance to antibiotics was reported as minimum inhibitory concentration (MIC) value or as susceptible, intermediate or resistant (SIR). We interpreted MICs according to the Clinical and Laboratory Standards Institute (CLSI) 2009 breakpoints [19]. The surveillance projects that reported SIR followed either CLSI or European Committee on Antimicrobial Susceptibility Testing (EUCAST) standards. We classified intermediately susceptible isolates as resistant. In addition, we used data from two publicly available country-level databases from the European Centre for Disease Prevention and Control’s (ECDC) European Antimicrobial Resistance Surveillance Network (EARS-Net) covering the years 1998 to 2014 [20] and the Center for Disease Dynamics, Economics and Policy (CDDEP) covering the years 1999 to 2015 [21]. A summary of the data included in each database is presented in Supplementary Table S1. We were unable to get access to two other large surveillance projects: the Study for Monitoring Antimicrobial Resistance Trends (SMART, Merck, Kenilworth, US) and the US CDC's National Healthcare Safety Network (NHSN).

We grouped all antibiotics into classes at the ATC4 level using the WHO’s Anatomical Therapeutic Chemical (ATC) classification method [22]. The one exception was the grouping of drugs used to define meticillin-resistant *Staphylococcus aureus* (MRSA): oxacillin and cefoxitin, which belong to different ATC groups but were grouped together to reflect diagnostic definitions [19]. An isolate was considered resistant to an antibiotic class if it was resistant to any drug in the class.

### Modelling antimicrobial resistance spread

We aggregated data at the country and year level to produce country–bacterium–antibiotic class triads (e.g. France–*Escherichia coli*–third-generation cephalosporins). For a triad to be included in the analysis, the following two criteria were required: resistance had to be reported on at least 100 isolates per year over at least 5 years (not necessarily consecutive). These criteria were set after examining the data, since lower number of isolates per year or shorter time periods resulted in high variability and did not allow accurate per-year and secular trend estimates.

We defined two states of spread of resistance: The first was ‘static’, i.e. no change in the resistance level was observed over time. A country was defined as static for a particular bacterium–antibiotic combination if: (i) the minimum resistance level was less than 2% and the maximum level did not exceed 2% or (ii) the minimum resistance level was between 2% and 15% and the maximum level was no more than 3 percentage points higher than the minimum level. The second state was ‘changing over time’, which included all the triads not in a static state. For these triads, we fit both linear and sigmoid models and chose the one with the better fit. A triad was considered to have a good fit if R^2^ (for linear models) or Efron's pseudo R^2^ (for sigmoid models) was ≥ 0.7 [23]. If neither of these models fit the data, that triad was classified as noise (no discernible pattern). We performed no further analyses on triads classified as static or noise.

Using the observed data, we generated sigmoid models; the models had four parameters (Formula 1) that were fit to the ‘changing over time’ triads.

Formula 1:

$$\%\;resistance\;(t)=\frac{X1}{1+e^{-X2(t-X3)}}+X4$$

X1 = resistance range in % (maximum − minimum resistance level)

X2 = sigmoid slope

X3 = year of 50% increase in resistance

X4 = initial resistance level in database

t = year

We assumed the following boundaries for the model parameters:

1. 0.02 ≤ X1 ≤ 1: the range of resistance was higher than 2% (otherwise, there was a static trend and the triad was excluded);
2. 0.3 ≤ X2 ≤ 3: we restricted the slope so as not to be too flat (i.e. a static trend) or too steep (i.e. an illogically rapid spread of resistance that suggests that there were errors in the data);
3. 1990 ≤ X3 ≤ 2012: based on our data, these were the relevant years to look for the start of the rise in resistance;
4. 0 ≤ X4 ≤ 0.2: the initial resistance level had to be less than 20% because a high initial resistance level would suggest that a previous rise had occurred in the past. Based on one author’s (YC) expert opinion, we assumed that an initial resistance level of up to 20% could have been the result of a slow rise over many years and our sigmoid model started after that unobserved initial rise.

For triads that fit sigmoid models but reached the maximum bound for the X2 parameter (X2 = 3, i.e. the rise in resistance was too steep), we examined whether the steep rise was the result of one outlying year by constructing several sigmoid models, each omitting 1 year. The best fitting model among those omitting 1 year was chosen if it did not cross the X2 bound. Otherwise, the original model with all years was used. The same procedure was used for triads that did not fit sigmoid models (pseudo R^2^ < 0.7): if the triad conformed to a sigmoid model after omitting 1 year, this was the model we used.

If a sigmoid model could not be fitted for a triad, a linear model was examined. If the R^2^ of the linear model was higher than 0.41, the triad was considered to have a linear trend over time. Linear models with positive or negative coefficients were considered to have a positive or negative trend, respectively. Triads with R^2^ ≤ 0.4 were classified as noise. In addition, all triads with sigmoid models were plotted and visually inspected. If the data were inconsistent with prior knowledge (e.g. a rapid drop in resistance), the triad was excluded.

### Grouping data by pace of resistance spread

For triads that conformed to a sigmoid model, the model slope was calculated. The model slope was the maximal sigmoid slope (i.e. the maximal rate of increase in resistance). This slope best described the pace of spread of resistance over time, and was calculated as X1 × X2/4. The calculated slope was used to classify triad progression into one of three characterising paces: slow, intermediate or fast. Pace category cut-offs were determined for each bacterium–antibiotic pair. Within each pace category, average sigmoid models (over countries) were calculated. Each of the three model parameters (X1, X2 and X4) was set to the average value of all countries in the category and X3 (value of middle year of slope, i.e. 50% increase) was set to 0 (in order to synchronise the maximal slope point).

### Validation

In order to validate the prediction in an iterative process, predicted and observed values were compared within each pace category for each triad, using the ‘take one out’ method. For example, to test the accuracy of our predictions for *E. coli*–third-generations cephalosporins in Country X in the fast spread category, we fitted an average model (as described above) using all the other countries in the fast category. Then, we calculated the absolute difference between the proportion of resistance predicted by the model and the observed proportion of resistance in Country X.

## Results

**Figure 1:**
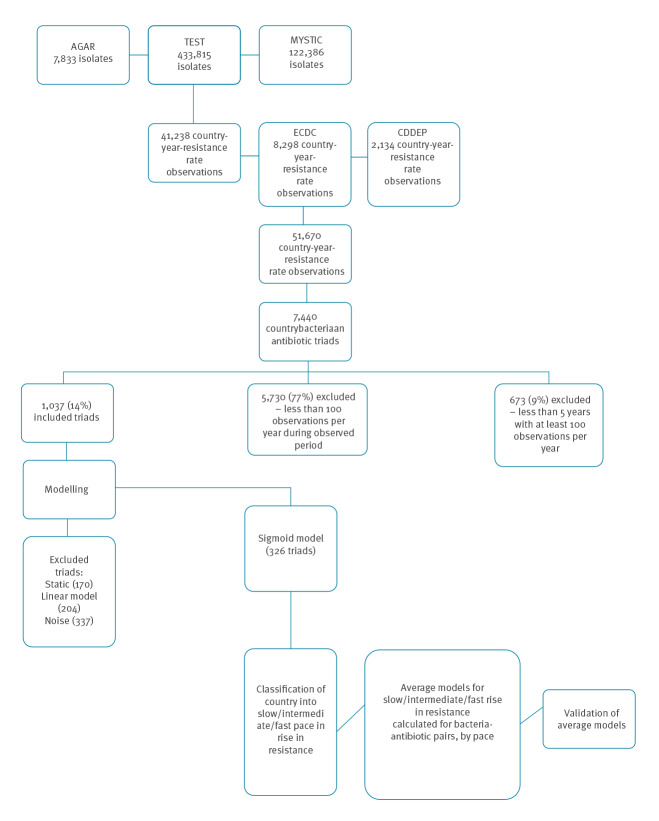
Data selection flowchart and overview of analysis of models to predict the spread of antimicrobial resistance, 1997–2015, 75 countries

AGAR: Australian Group on Antimicrobial Resistance; CDDEP: Center for Disease Dynamics, Economics and Policy; ECDC: European Centre for Disease Prevention and Control; MYSTIC: Meropenem Yearly Susceptibility Test Information Collection.

Our database included isolate-level data on 510,297 isolates and country-level data on 23 years from 71 countries. We grouped the data into 51,670 observations of country–year–bacterium–antibiotic which included 30 bacterial species and 54 antibiotics in 25 classes (Figure 1). These observations were grouped into 7,440 country–bacterium–antibiotic triads. Of the triads, 5,730 had less than 100 isolates per year, and 673 had less than 5 years of data and were therefore considered non-informative and excluded from further analysis. Thus, 1,037 triads (14%) met the inclusion criteria and formed our analytic sample. An overview of the analysis is presented in Figure 1.

Number of countries covered by area: Europe (n = 34), Asia (n = 17), South America: (n = 6), Africa (n = 6), Central America (n = 6), North America (n = 2), Oceania (n = 2), Caribbean (n = 2).

When modelling the pattern over time for the 1,037 triads, 700 triads were informative. Table 1 presents the distribution of spread patterns (details in Supplementary Figure S2 and Supplementary Table S3). A third of the triads (n = 326; 31.4%) followed a sigmoid (logistic) pattern over time. Among 107 triads for which both a sigmoid and linear models could be fitted, the sigmoid model was better in 90 (84%). 

**Table 1:** Patterns of antimicrobial resistance spread, 1997–2015

Category	All country-bacterium-antibiotic triads^a^		Selected bacterium–antibiotic pairs**^b^**															
			Carbapenem-resistant *Pseudomonas aeruginosa*		Carbapenem-resistant *Acinetobacter baumannii*		Meticillin-resistant *Staphylococcus aureus*		Third-generation cephalosporin-resistant* Klebsiella pneumoniae*		Carbapenem-resistant *Klebsiella pneumoniae*		Fluoroquinolone-resistant* Escherichia coli *		Third-generation cephalosporin-resistant *Escherichia coli *		Carbapenem-resistant *Escherichia coli *	
	n	%	n	%	n	%	n	%	n	%	n	%	n	%	n	%	n	%
Sigmoid	326	31	9	33	5	63	8	23	18	56	10	32	32	82	28	74	2	5
No discernible pattern	337	32	12	44	2	25	13	37	9	28	3	10	3	8	8	21	7	18
Static	170	16	0	0	0	0	3	9	0	0	14	45	0	0	0	0	25	66
Positive linear trend	55	5	3	11	1	13	1	3	4	13	1	3	2	5	1	3	1	3
Negative linear trend	149	14	3	11	0	0.0	10	29	1	3	3	10	2	5	1	3	3	8
**All**	**1,037**	**100**	**27**	**100**	**8**	**100**	**35**	**100**	**32**	**100**	**31**	**100**	**39**	**100**	**38**	**100**	**38**	**100**

^a ^Numbers in rows do not sum up to all countries because the columns are selected examples of bacteria–antibiotic pairs, not all pairs.

^b ^The numbers in each cell refer to the number of countries whose data fit a given pattern. For example, the spread of carbapenem resistance among *P. aeruginosa* followed a sigmoid pattern in nine countries.

**Figure 2:**
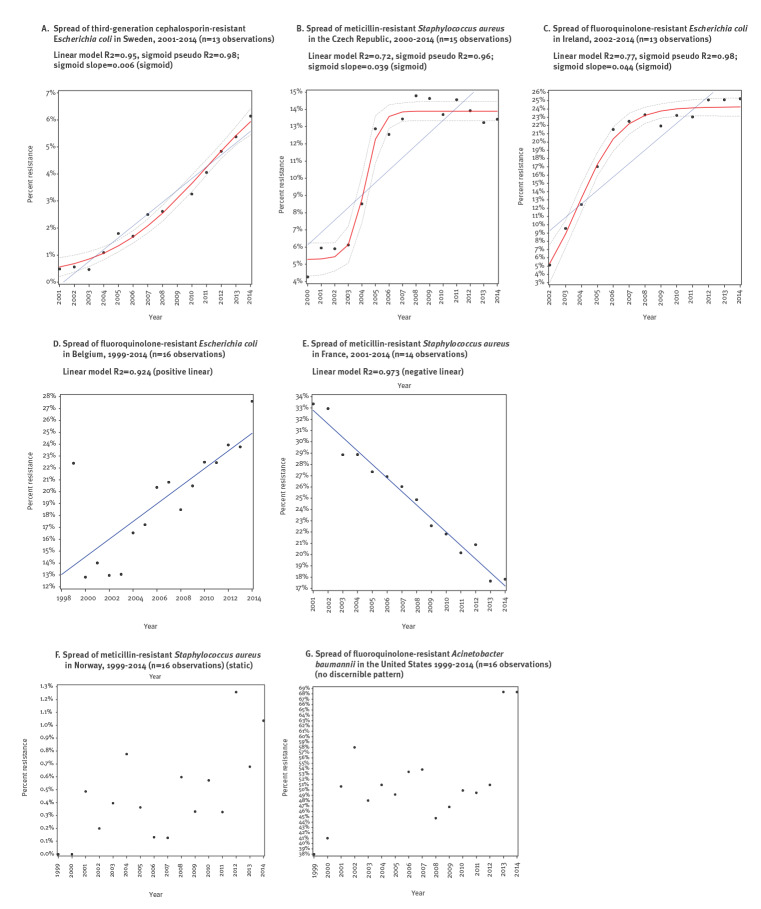
Examples of patterns of antimicrobial resistance spread

Figures 2A to 2C present examples of triads for which the sigmoid model was best. Fifty-five triads (5.3%) followed a positive linear pattern (Figure 2D), 149 (14.4%) followed a negative linear pattern (Figure 2E) and 170 (16.4%) were static (Figure 2F). The remaining 337 triads (32.5%) followed no discernible pattern (Figure 2G).

Black dots: observed proportion of resistance; red lines: sigmoid model; dotted lines: 95% confidence bands. Blue line: linear regression line best fitted to the data.

Among 381 triads in which resistance was rising, in 326 (85.6%) a sigmoid pattern explained the spread. To generate an average rate of spread for each bacterium–antibiotic class pair, we required the sigmoid models of at least four countries. Of the 326 triads, sigmoid models from at least four countries were available for 259 triads (representing eight bacteria and 23 bacterium–antibiotic class pairs) (Table 2 and Supplementary Table S4). The bacteria in the majority of these triads (181/259, 69.9%) were *E. coli* and *Klebsiella pneumoniae*.

**Table 2:** Bacterium–antibiotic pairs classified by rate of resistance spread (n = 259 triads modelled as sigmoid)

Bacterium	Antibiotic class	Slow		Intermediate		Fast		Countries (n)
		n	% of countries	n	% of countries	n	% of countries	
*Escherichia coli*	Third-generation cephalosporins	13	46	8	29	7	25	28
	Quinolones	24	75	4	13	4	13	32
	Aminoglycosides	17	63	5	19	5	19	27
	Penicillins	8	57	4	29	2	14	14
	BL/BLI	5	50	2	20	3	30	10
*Klebsiella pneumoniae*	Third-generation cephalosporins	5	28	3	17	10	56	18
	Carbapenems	4	40	4	40	2	20	10
	Aminoglycosides	13	62	6	29	2	10	21
	BL/BLI	2	25	3	38	3	38	8
	Quinolones	4	31	5	38	4	31	13
*Staphylococcus aureus*	Oxacillin	1	13	6	75	1	13	8
	Rifampicin	2	33	2	33	2	33	6
*Acinetobacter baumannii*	Carbapenems	2	40	2	40	1	20	5
	Third-generation cephalosporins	0	0	3	75	1	25	4
*Enterococcus faecium*	Glycopeptides	3	50	2	33	1	17	6
	Aminoglycosides	2	33	2	33	2	33	6
	Penicillins	4	57	1	14	2	29	7
*Pseudomonas aeruginosa*	Carbapenems	2	22	7	78	0	0	9
	Piperacillin-tazobactam	4	44	2	22	3	33	9
	Quinolones	1	25	2	50	1	25	4
*Streptococcus pneumoniae*	Penicillins	4	67	2	33	0	0	6
	Macrolides	4	100	0	0	0	0	4
*Enterobacter*	Carbapenems	2	50	2	50	0	0	4

BL: beta-lactam; BLI: beta-lactamase inhibitor.

Based on visual inspection of country-specific graphs, 126 (48.6%) triads were categorised as having a slow spread of resistance, 77 (29.7%) as intermediate spread, and 56 (21.6%) as rapid spread. For each bacterium–antibiotic class pair, an average sigmoid model was calculated for countries with slow, intermediate and rapid spread. For example, we calculated the average pattern of spread of third-generation cephalosporin-resistant *E. coli* in countries with a slow, intermediate or fast increase in resistance (Supplementary Figure S5). Starting at 100% susceptibility, in a country with a typically slow spread of resistance, based on our models we could expect a level of 10% resistance after 20 years. In contrast, in countries with fast patterns of spread, we could expect almost 30% resistance within 7 years.

**Figure 3:**
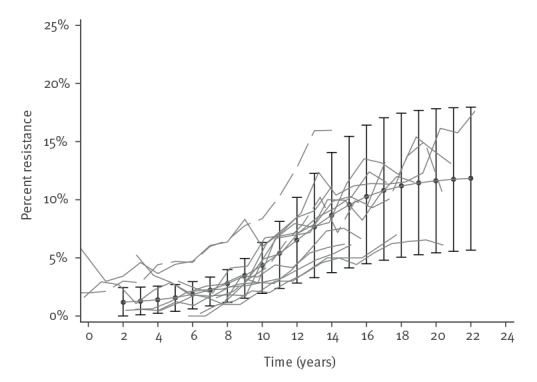
Slow spread of third-generation cephalosporin resistance in *Escherichia coli,* 13 countries

In order to validate the average models, we compared data on observed country-specific resistance rates with rates predicted by the model. Figure 3 presents observed data on third-generation cephalosporin resistance in *E. coli* from 13 countries in the ‘slow’ category over 22 years. Superimposed on these is our calculated average model for this ‘slow’ category (additional examples in Supplementary Figure S6 a-d). Overall, the median difference between observed and predicted values was 6.5% (interquartile range: 2.8–12.6%), with lower median difference for countries with a slow spread of resistance and greater differences for countries with intermediate and fast spread (Supplementary Table S7).

Average model with confidence intervals and individual country graphs.

Grey dashed lines: observed third-generation cephalosporin resistance in *E. coli* over time of in 13 countries. Black dots: predicted resistance; interval bars: the 95% confidence intervals. All individual country lines were calibrated to a numeric scale for comparison (i.e. the first year of data was called Year 1), each unit representing 1 year.

## Discussion

In this study, we assembled a database of AMR from most of the existing large surveillance projects. Exploring these data, we found support for our hypothesis that sigmoid models describe the spread of AMR more accurately than linear models in most cases. We presented new methods for modelling the spread of resistant organisms based on country-specific dynamics. For countries with sufficient surveillance data, in which resistance in a given antibiotic–bacterium pair changed over time, we were able to classify the spread of resistance into a slow, intermediate or fast pace. Using these methods, we were able to predict the future spread of AMR in a given country based on historical observations.

Most previous models and predictions of the spread of resistance have assumed linear progression (although a very early study of resistance among staphylococci hinted at a sigmoid pattern of spread [24]). At the early stages of emergence of resistance, there are few carriers of AMR who serve as the reservoir for transmission; thus, few new cases occur. Later, when carriage is more prevalent, transmission enters a growth phase. Finally, spread reaches a plateau either when the susceptible population has been exhausted or when the number of new acquisitions of AMR equals the number of carriers who recover from carriage. The slope of the growth phase and the level at which the plateau occurs are closely related; both depend on the ease of transmission in a specific location and time. For example, in countries with good sanitation, spread of AMR by the faecal-oral route will be slower and will plateau at a lower level than in countries with poor sanitation. A similar pattern will occur among AMR bacteria which spread within hospitals in countries with healthcare systems with good infection control practices. Notably, the levelling off of spread of resistance represented by the sigmoid model is in contrast to the linear model in which resistance rises indefinitely.

In our dataset, although most country–bacterium–antibiotic triads with a discernible trend followed a sigmoid pattern, some triads followed a positive or negative linear pattern. We believe that the positive linear patterns represent the growth phase of the sigmoid pattern in which the initial phase has been censored, i.e. it occurred before data were collected. We assume that the negative linear trends represent either clone fatigue (i.e. the clonal fitness cost of resistance exceeded its evolutionary advantage) or infection control interventions that successfully reversed the spread of resistance [25,26].

Modelling of AMR and predictions of future prevalence contributes considerably to public health policy [27]. Our finding of mostly sigmoidal spread of AMR, in which there is a close correlation between the slope of the growth phase and the level at which resistance reaches a steady state, has important implications for policymakers. In countries with fast spread, the plateau will occur at a higher level of resistance. Therefore, interventions to slow the spread of resistance have a long-term impact and will translate into lower levels of resistance in the future. For AMR that spreads primarily in the community, important factors influencing spread include: sanitation and hygiene, especially the separation of sewage from the water supply, living conditions and crowding, vaccination and antibiotic use as well as food safety. For AMR that is primarily healthcare-associated, important factors are: hand hygiene, patient–staff ratios, antibiotic use, environmental cleanliness and interconnectedness of healthcare facilities. Thus, the pace of spread is multifactorial and may change over time. Interventions on a country level can successfully reverse the spread of resistance [28-30]. On the other hand, emergence of new clones and/or mechanisms of resistance may lead to an upsurge in AMR. It is important to note that our models assume that no intervention has taken place to limit the spread of resistance and no new clone has arisen. If country level interventions had an important impact, one would expect a reduction in resistance, an uncommon phenomenon in our data. We hypothesise that the emergence of a successful new clone will reset the model and a superimposed sigmoid will arise.

Our findings also have implications for researchers interested in assessing the impact of interventions to slow the spread of antibiotic resistance or predicting future resistance. Assuming a linear model may lead to erroneous interpretations of the results of interventions; plateauing of resistance levels will be interpreted as success when in fact the plateau is the natural course of the sigmoid model. Using a sigmoid model, a successful intervention should appear as a reduction in resistance levels. Preferably, local data should be used to determine the shape and pace of spread in the region of interest. If data are not available, a sigmoid progression should be assumed, the category (slow, intermediate or fast) should be chosen based on prior knowledge and the average pace for that category should be applied. We have produced such country-level estimates based on the best publicly available data (http://www.epi-ar.org/). The sigmoid models and predictions can be updated yearly as data accumulate.

Our study reveals the shortcomings of existing surveillance projects; although we had data from most large AMR surveillance projects, only a fraction of the data were informative: i.e. there was continuity over time from the same country and the number of bacterium–antibiotic pairs was sufficient to avoid fluctuation by chance alone. To allow prediction and modelling of AMR based on surveillance data, we recommend including at least 100 antibiotic–bacterium pairs per year and continuity of at least 5 years in a given country. Other recommendations for improvement of surveillance have been described previously [13].

Our study has several limitations. Firstly, as noted earlier, although we assembled a large dataset of AMR isolates, only a small subset was usable, mainly from high-income countries. There were not enough data from low- and middle-income countries on which to validate our models. Secondly, the sigmoid models, which performed best to explain changes in AMR overtime, still explained only a third of the dynamics of AMR spread. Thirdly, surveillance projects, and therefore our methods, miss the earliest stages of AMR spread, the stages at which interventions are most successful. Lastly, modelling of AMR spread is inherently limited by the same chance elements that affect all long-term predictions in infectious disease epidemiology [31]. All these limitations combined suggest that predictions of future spread of resistance worldwide carry a high degree of uncertainty.

## Conclusion

We present a novel method of describing and predicting the spread of antibiotic-resistant organisms. Our results can help identify countries at risk of rapid spread of resistance and may be used to inform policy. We encourage investing in high-quality surveillance systems, enabling more accurate future predictions.

## Supplementary Data

753000 Supplement
