# Supplementary material for: Utilising sigmoid models to predict the spread of antimicrobial resistance at the country level
Source: Euro Surveill. 2020 Jun 11;25(23):1900387. doi: 10.2807/1560-7917.ES.2020.25.23.1900387 (PMC7403637; doi:10.2807/1560-7917.ES.2020.25.23.1900387)
Supplement: Supplement [file 19-00387_FALLACH_Supplement.pdf]

Supplementary material for Fallach *et al.*, "Utilising sigmoid models to predict the spread of antimicrobial resistance at the country level"

This supplementary material is hosted by Eurosurveillance as supporting information alongside the article "Utilising sigmoid models to predict the spread of antimicrobial resistance at the country level" on behalf of the authors, who remain responsible for the accuracy and appropriateness of the content. The same standards for ethics, copyright, attributions and permissions as for the article apply. Supplements are not edited by Eurosurveillance and the journal is not responsible for the maintenance of any links or email addresses provided therein.

Table S1: Summary of data included in each database

Database name (sponsor)	AESOP, EnSOP (Australian Group on Antimicrobial Resistance)	CDDEP	EARS-Net (ECDC)	TEST (Pfizer)	MYSTIC (AstraZeneca)
**Observations**	7833	2134	8298	313596	122386
**Years**	2013	Specific	Specific	2004-2014	1997, 1999, 2000-2007
**Country**	Australia	Global	Europe	Global	Global
**N. Bacteria**	19	10	8	18	29
**N. Antibiotics**	35	10	10	19	24
**Age**	Not specified	Not specified	Not specified	Available	Available
**Specimen source**	Blood	Blood, CSF	Blood, CSF	Various	Various
**Date collected**	Exact	Year	Year	Exact	Exact
**Patient population**	Inpatients	Inpatients and outpatients	Inpatients	Inpatients	Inpatients
**Acquisition (hospital or community)**	Specified	Not specified	Not specified	Not specified	Not specified

Figure S2: Graphical presentation of *E.coli* resistance to 3rd-generation cephalosporins at the country level, by model type

Some graphs that appear linear (for example, South Africa) have been classified as sigmoid. As seen in Supplementary Table S4, the R^2 was higher for the sigmoid model than for the linear model.

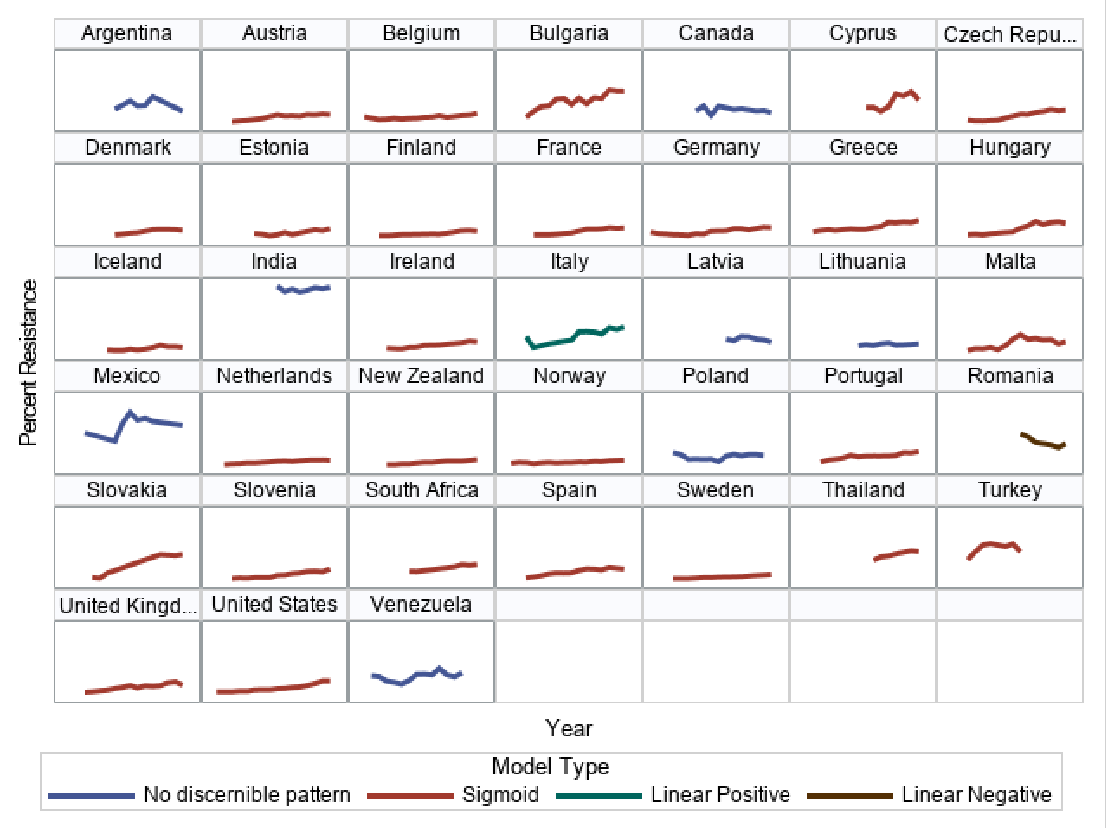

Table S3: *E.coli* resistance to 3rd-generation cephalosporins at the country level: comparison of R^2 for the sigmoid and linear models

In 3 cases (Bulgaria, New Zealand and Portugal) the R^2 for the linear model was slightly higher than for the sigmoid model, but we determined *a priori* that any country whose sigmoid model had an R^2 greater than 0.7 would be classified as sigmoid.

Country	Model Type	Sigmoid R^2	Linear R^2
Argentina	No discernible pattern		
Austria	Sigmoid	0.95	0.86
Belgium	Sigmoid	0.76	0.13
Bulgaria	Sigmoid	0.78	0.79
Canada	No discernible pattern		
Cyprus	Sigmoid	0.84	0.55
Czech Republic	Sigmoid	0.98	0.93
Denmark	Sigmoid	0.96	0.80
Estonia	Sigmoid	0.83	0.65
Finland	Sigmoid	0.93	0.89
France	Sigmoid	0.99	0.93
Germany	Sigmoid	0.88	0.69
Greece	Sigmoid	0.95	0.89
Hungary	Sigmoid	0.97	0.88
Iceland	Sigmoid	0.86	0.68
India	No discernible pattern		
Ireland	Sigmoid	0.96	0.95
Italy	Linear Positive		0.74
Latvia	No discernible pattern		
Lithuania	No discernible pattern		
Malta	Sigmoid	0.83	0.49
Mexico	No discernible pattern		
Netherlands	Sigmoid	0.97	0.95
New Zealand	Sigmoid	0.97	0.98
Norway	Sigmoid	0.90	0.76
Poland	No discernible pattern		
Portugal	Sigmoid	0.83	0.86
Romania	Linear Negative		0.75
Slovakia	Sigmoid	0.99	0.96
Slovenia	Sigmoid	0.97	0.94
South Africa	Sigmoid	0.99	0.10
Spain	Sigmoid	0.98	<0.01
Sweden	Sigmoid	0.98	0.97
Thailand	Sigmoid	0.98	0.92
Turkey	Sigmoid	0.77	0.21
United Kingdom	Sigmoid	0.87	0.85
United States	Sigmoid	0.98	0.89
Venezuela	No discernible pattern		

Table S4: List of countries belonging to each rate of resistance spread, by bacterium-antibiotic pair

Bacterium	Antibiotic class	Rate of resistance spread	Countries
Acinetobacter baumannii	Carbapenem	Slow	France, United States
		Intermediate	Italy, Venezuela
		Fast	Mexico
	Cephalosporins 3	Intermediate	France, United States, Venezuela
		Fast	Mexico
Enterobacter	Carbapenem	Slow	Canada, United States
		Intermediate	Thailand, Venezuela
Enterococcus faecium	Aminoglycosides	Slow	Austria, Italy
		Intermediate	Netherlands, Sweden
		Fast	Czech Republic, Spain
	Glycopeptide	Slow	Austria, Denmark, Norway
		Intermediate	Hungary, Ireland
		Fast	Venezuela
	PNC	Slow	Austria, Norway, Spain, Sweden
		Intermediate	Czech Republic
		Fast	France, Netherlands
Escherichia coli	Aminoglycosides	Slow	Argentina, Austria, Belgium, Czech Republic, Denmark, Finland, France, Hungary, Ireland, New Zealand, Portugal, Slovakia, Slovenia, Spain, Sweden, Thailand, United Kingdom
		Intermediate	Estonia, Greece, Iceland, Italy, Luxembourg
		Fast	Cyprus, India, Romania, South Africa, Turkey
	Cephalosporins 3	Slow	Belgium, Finland, Germany, Ireland, Netherlands, New Zealand, Norway, Portugal, Slovenia, Spain, Sweden, United Kingdom, United States
		Intermediate	Austria, Bulgaria, Czech Republic, Denmark, Estonia, France, Greece, Iceland
		Fast	Cyprus, Hungary, Malta, Slovakia, South Africa, Thailand, Turkey
	PNC	Slow	Belgium, Czech Republic, France, Italy, Poland, Spain, United Kingdom, United States
		Intermediate	Argentina, Austria, Ireland, Malta
		Fast	Portugal, South Africa
	PNC/Bli	Slow	Belgium, Germany, Spain, Thailand, United States
		Intermediate	Italy, Mexico
		Fast	India, South Africa, Turkey
	Quinolone	Slow	Austria, Bulgaria, Czech Republic, Denmark, Estonia, Finland, France, Germany, Greece, Hungary, Ireland, Italy, Luxembourg, Netherlands, New Zealand, Norway, Poland, Slovenia, Spain, Sweden, Thailand, United Kingdom, United States, Venezuela
		Intermediate	Lithuania, Portugal, South Africa, Turkey

		Fast	Argentina, Cyprus, Malta, Slovakia
Klebsiella pneumoniae	Aminoglycosides	Slow	Belgium, Canada, Croatia, Czech Republic, Denmark, Finland, France, Hungary, New Zealand, Portugal, Slovenia, Spain, Sweden
		Intermediate	Argentina, Bulgaria, Greece, Italy, Malta, South Africa
		Fast	India, Poland
	Carbapenem	Slow	Belgium, Hungary, Spain, Thailand
		Intermediate	Argentina, Greece, United States, Venezuela
		Fast	India, Italy
	Cephalosporins 3	Slow	Netherlands, New Zealand, Spain, Sweden, United States
		Intermediate	Austria, France, South Africa
		Fast	Bulgaria, Croatia, Czech Republic, Greece, Ireland, Italy, Malta, Portugal, Slovenia, Thailand
	PNC/Bli	Slow	Belgium, United States
		Intermediate	Mexico, New Zealand, Spain
		Fast	France, Italy, South Africa
	Quinolone	Slow	France, Mexico, Slovenia, Spain
		Intermediate	Austria, Greece, Italy, Malta, Portugal
		Fast	Czech Republic, Hungary, South Africa, Thailand
MRSA	Oxacilin	Slow	Finland
		Intermediate	Canada, Czech Republic, Hungary, Portugal, Romania, Slovakia
		Fast	Venezuela
Pseudomonas aeruginosa	Carbapenem	Slow	Hungary, Spain
		Intermediate	France, Greece, Mexico, Poland, Slovenia, Sweden, Venezuela
	Piperacillinntaz	Slow	Norway, Portugal, Sweden, United Kingdom
		Intermediate	Austria, Ireland
		Fast	Belgium, Italy, Slovenia
	Quinolone	Slow	Portugal
		Intermediate	Spain, Venezuela
		Fast	Mexico
Staphylococcus aureus	Rifampicin	Slow	Malta, Slovakia
		Intermediate	Bulgaria, Lithuania
		Fast	Finland, Portugal
Streptococcus pneumoniae	Macrolide	Slow	Finland, Ireland, United States, Venezuela
	PNC	Slow	Denmark, Finland, Norway, Sweden
		Intermediate	Germany, Ireland

Figure S5: Average models of rise in resistance over time for the three categories of *E.coli* resistance to 3rd-generation cephalosporins

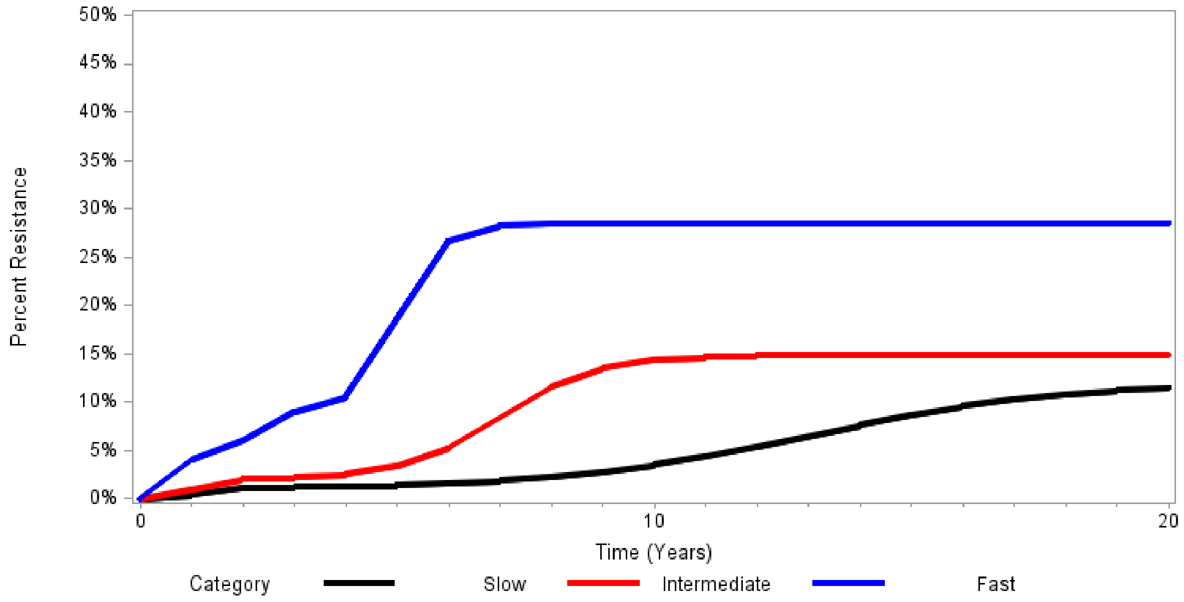

Figures S6 a-d: Average model with confidence intervals and individual country graphs

Grey dashed lines represent observed resistance rate over time. Black dots represent predicted resistance rate, with interval bars showing the CI of prediction. All individual country lines were calibrated to a numeric scale for comparison, each unit presenting 1 year.

a. *E.coli* resistance to 3rd-generation cephalosporins with intermediate pace (comparison of average model to observed data from 7 countries)

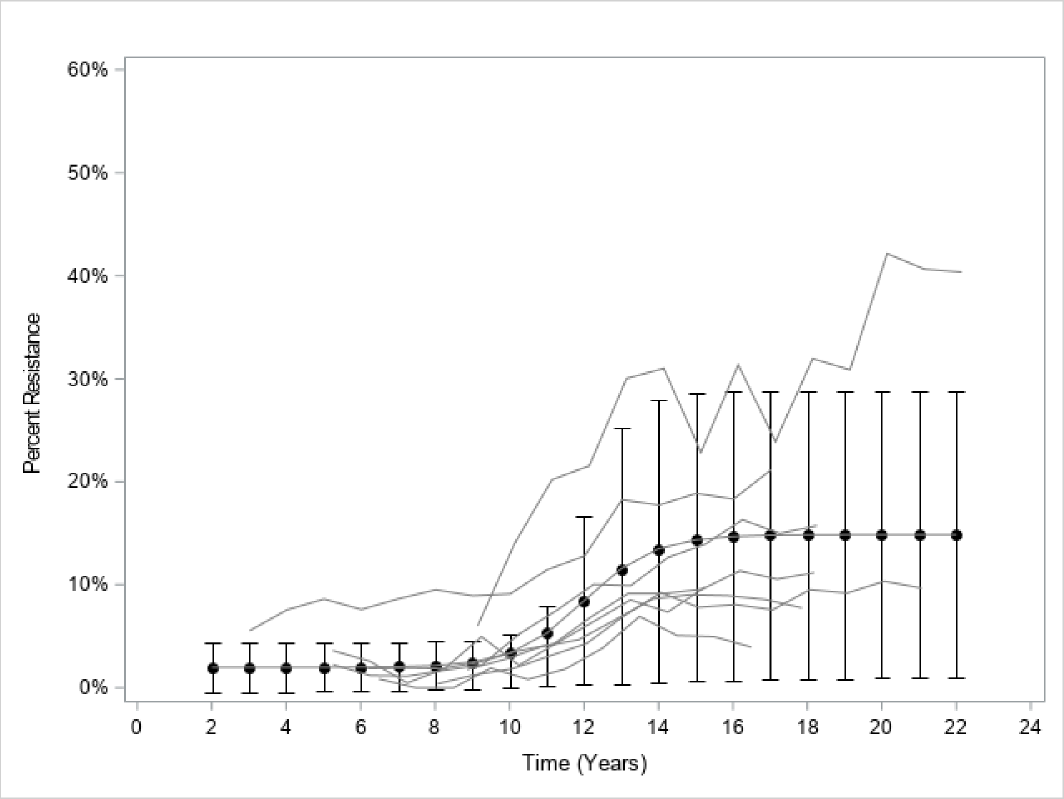

b. *E.coli* resistance to 3rd-generation cephalosporins with fast pace (comparison of average model to observed data from 7 countries)

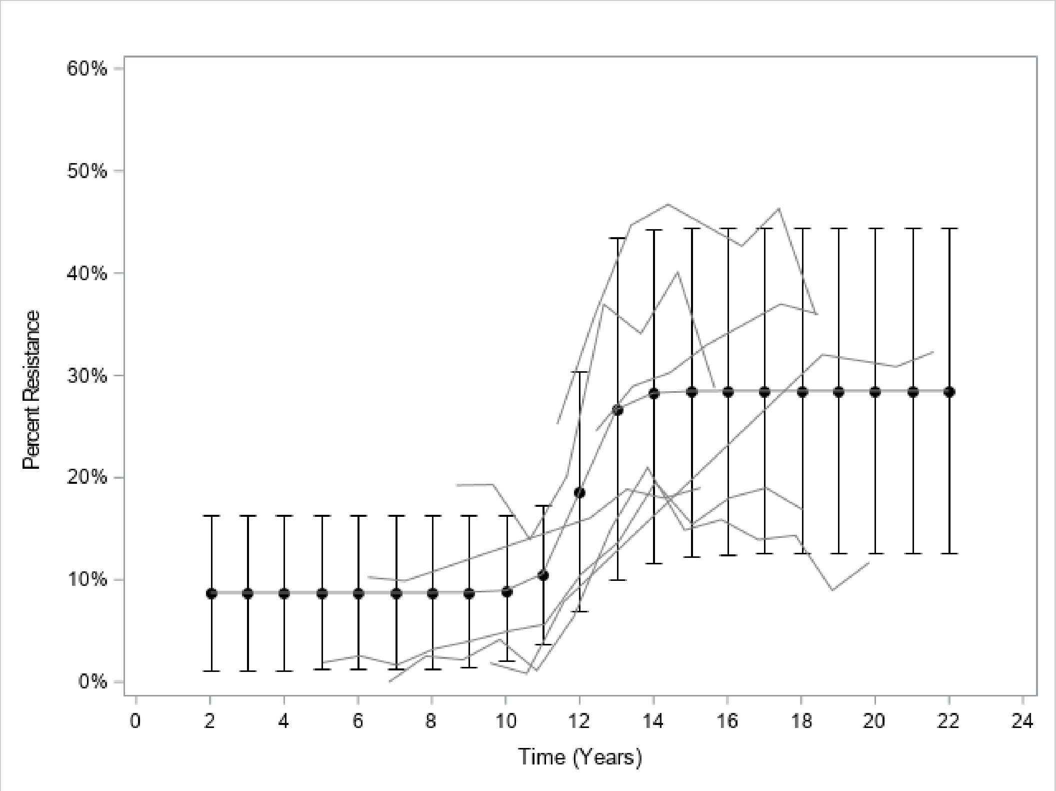

c. *Klebsiella Pneumonia* resistance carbapenems with slow pace (comparison of average model to observed data from 5 countries)

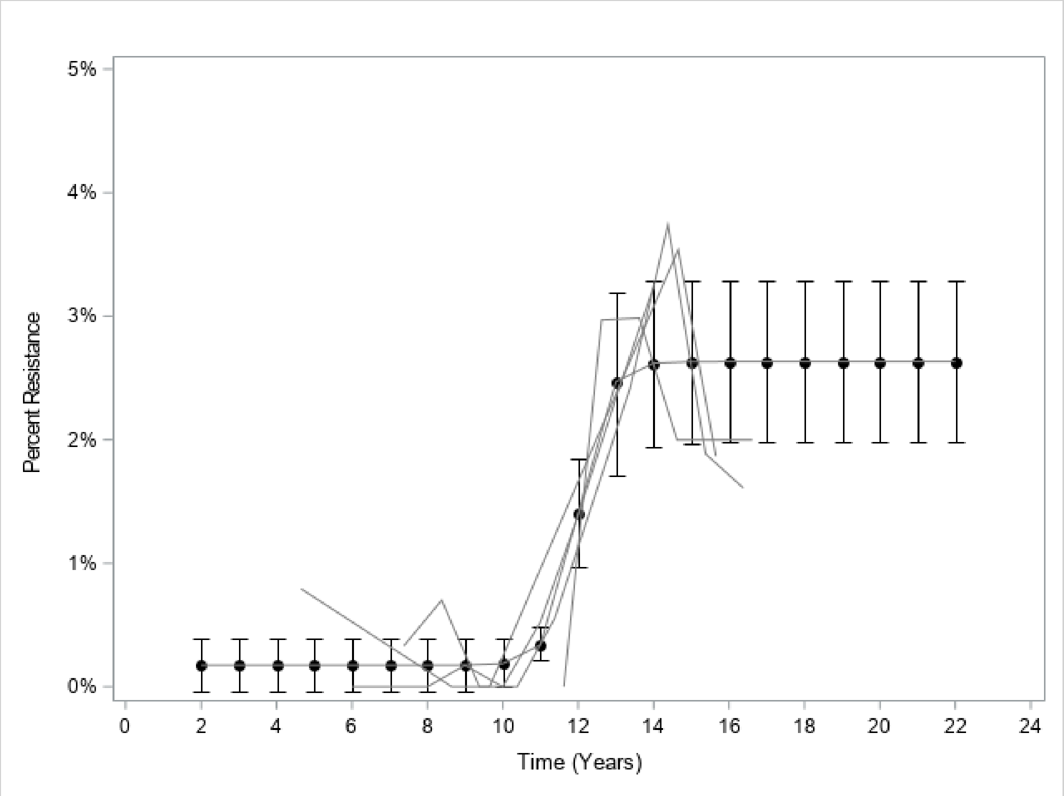

d. MRSA with intermediate pace (comparison of average model to observed data from 6 countries)

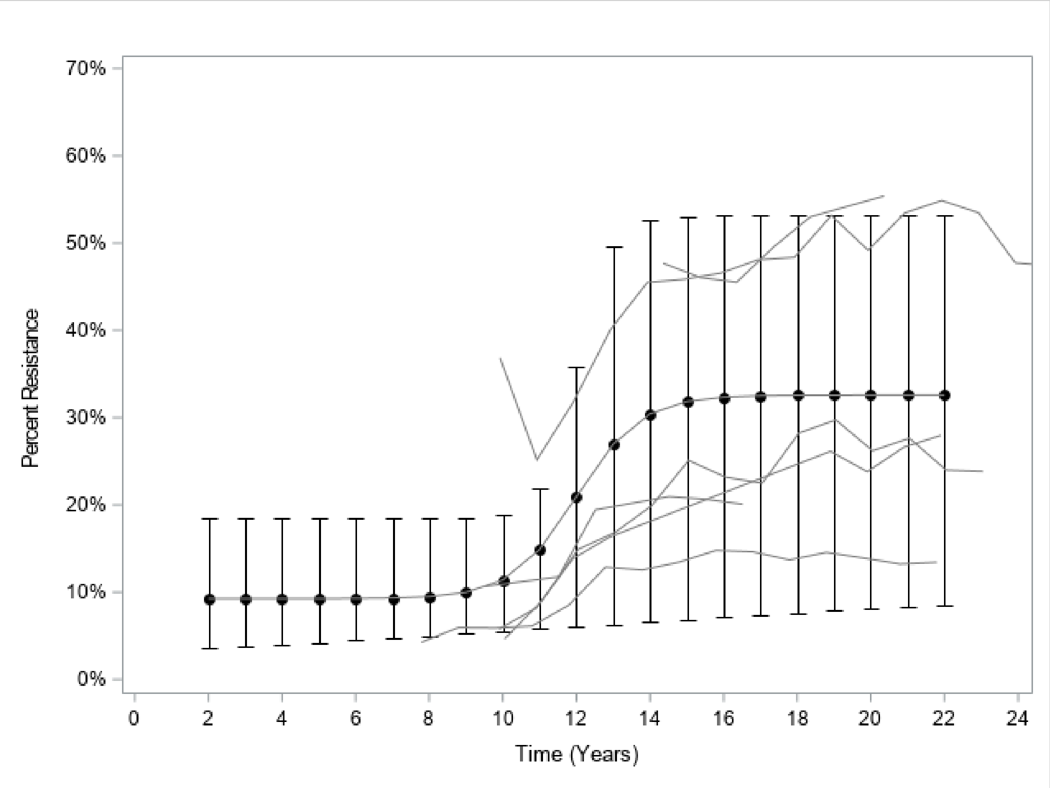

Table S7: Absolute difference between observed and predicted proportion of resistance

Rate of resistance spread	25th percentile	Median	75th percentile	N
**Slow**	2.0%	4.1%	8.0%	785
**Intermediate**	5.3%	10.2%	18.5%	373
**Fast**	7.7%	13.5%	20.6%	223
**All**	2.8%	6.5%	12.6%	1381
